# Associations among sleep quality, cognitive decline, and Alzheimer's disease pathology in older adults: A longitudinal study

**DOI:** 10.1002/alz.71470

**Published:** 2026-06-03

**Authors:** Layaly Shihadeh, Mónica Rosselli, Joshua Conniff, Breton Asken, Alicia Goytizolo, Warren Barker, Emily F. Matusz, Angel Collie, Stephen A. Coombes, Melissa Armstrong, Idaly Velez‐Uribe, Malek Adjouadi, Glenn E. Smith, Ranjan Duara, Steven D. DeKosky, David A. Loewenstein

**Affiliations:** ^1^ Department of Psychology Charles E. Schmidt College of Science, Florida Atlantic University Davie Florida USA; ^2^ 1Florida Alzheimer's Disease Research Center Miami Beach and Gainesville Florida USA; ^3^ Department of Clinical and Health Psychology College of Public Health and Health Professions, University of Florida Gainesville Florida USA; ^4^ Wien Center for Alzheimer's Disease and Memory Disorders Mount Sinai Medical Center Miami Beach Florida USA; ^5^ Department of Applied Physiology and Kinesiology University of Florida Gainesville Florida USA; ^6^ Department of Neurology University of Florida Gainesville Florida USA; ^7^ Center for Advanced Technology and Education College of Engineering, Florida International University Miami Florida USA; ^8^ Department of Psychiatry and Behavioral Sciences and Center for Cognitive Neuroscience and Aging Miller School of Medicine, University of Miami Miami Florida USA

**Keywords:** amyloid beta, cognitive impairment, hippocampal volume, Pittsburgh Sleep Quality Index, phosphorylated tau217

## Abstract

**INTRODUCTION:**

This study aimed to investigate whether sleep quality predicts cognitive/functional decline, and whether Alzheimer's disease (AD) pathology modifies these relationships.

**METHODS:**

The Pittsburgh Sleep Quality Index was administered to 326 older adults (113 cognitively normal, 192 mild cognitive impairment, 21 dementia; mean age = 66.4 ± 8.0 years) enrolled in the 1Florida Alzheimer's Disease Research Center. The Clinical Dementia Rating Sum of Boxes (CDR‐SB) assessed cognitive/functional decline at baseline and over time. Moderators included hippocampal volume (HV), amyloid beta (Aβ) positron emission tomography, and plasma phosphorylated tau (p‐tau)217.

**RESULTS:**

Cross‐sectionally, longer sleep duration and later wake time were associated with worse CDR‐SB, with stronger associations observed among individuals with higher Aβ and p‐tau217 levels, and smaller HV. Longitudinally, prolonged sleep duration was associated with faster cognitive decline, particularly in individuals with elevated Aβ or p‐tau217 levels and smaller hippocampal volumes at baseline.

**DISCUSSION:**

Prolonged sleep duration and later wake times predicted worsening cognitive performance, and these effects were strengthened by greater AD pathology.

## Highlights

- Longer sleep duration and later wake times were associated with higher Clinical Dementia Rating Sum of Boxes scores at baseline and over time.
- The presence of amyloid and phosphorylated tau217 pathology, as well as reduced hippocampal volume, potentiates the effect of sleep disturbances on cognitive/functional impairment.
- Results suggest that sleep measures may serve as early markers (before cognitive impairment is observed) and potential targets for intervention in cognitive decline associated with aging and Alzheimer's disease.

## 1 BACKGROUND

Sleep is essential to overall well‐being, as it influences mood, physical and mental health, and cognition. Sleep disturbances are common in older adults, with ≈ 40% of individuals worldwide reporting poor sleep quality,^1^ and up to 43% to 50% experiencing difficulty initiating or maintaining sleep.^2^ Age‐related changes in sleep architecture often lead to reduced sleep quality, contributing to declines in overall health, particularly cognitive functioning.^3^ As the aging population and prevalence of Alzheimer's disease (AD) increase, understanding the relationship between sleep quality and cognitive decline is critical for early detection and intervention.

Prior research has demonstrated associations between sleep quality and cognitive performance in older adults. Poor sleep quality has been linked to declines in reasoning, executive function, and semantic fluency.^4^, ^5^ Longitudinal findings suggest that longer sleep duration (> 8 hours) may also be associated with cognitive decline over time.^6^ Additionally, observable nighttime behaviors have been shown to align closely with AD biomarkers.^7^ Consistent with these findings, sleep disturbances are more pronounced in individuals with clinical cognitive impairment. Yu et al.^8^ reported that multiple sleep domains, including duration, disturbances, latency, efficiency, quality, and daytime dysfunction measured by the Pittsburgh Sleep Quality Index (PSQI), were significantly worse in individuals with mild cognitive impairment (MCI) compared to cognitively healthy adults.

Beyond cognitive and behavioral outcomes, growing evidence links sleep disturbances to AD pathology. Longer sleep latency has been associated with a higher amyloid beta (Aβ) burden in cognitively healthy older adults,^9^ and poor sleep quality is linked to abnormal brain microstructure, including lower global white matter restricted isotropic diffusion.^10^ Reduced slow‐wave sleep, often considered the most restorative sleep stage, has also been linked to accelerated atrophy in medial temporal lobe regions, including the hippocampus.^11^, ^12^ Additionally, both subjectively reported sleep problems and objectively measured sleep fragmentation were associated with higher plasma concentrations of phosphorylated tau at threonine 217 (p‐tau217), a blood‐based marker of AD pathology.^13^ Collectively, these findings suggest that sleep disturbances may be mechanistically involved in the progression of neurodegenerative processes. One proposed mechanism is impaired glymphatic clearance during sleep, which may reduce the removal of Aβ and tau proteins, thereby facilitating their accumulation over time.^12^

Despite this growing body of literature on sleep and cognition, important gaps remain. First, much of the existing research is cross‐sectional, limiting conclusions about directionality or whether sleep disturbances predict future cognitive/functional decline. Second, although associations between sleep and individual AD biomarkers have been established, minimal research has examined whether AD biomarkers moderate the relationship between sleep quality and clinical measures of disease severity, such as the Clinical Dementia Rating Sum of Boxes (CDR‐SB), a widely used measure of cognitive and functional impairment across Alzheimer's Disease Research Centers (ADRCs) in the United States. Third, most studies rely on PSQI global scores, with limited investigation into whether specific sleep domains (e.g., latency, efficiency, disturbances) show distinct associations with cognitive outcomes or disease progression.

The aim of the present study was to examine associations between PSQI global and subscale scores and CDR‐SB scores both cross‐sectionally and longitudinally. The study also evaluated whether AD biomarkers, including hippocampal volume, amyloid burden, and plasma p‐tau217, moderate these associations. It was hypothesized that greater cognitive and functional impairment would be associated with poorer sleep quality at baseline, and that worse baseline sleep quality would predict steeper increases in CDR‐SB scores over time. Additionally, it was predicted that these relationships would be moderated by AD biomarkers, such that lower hippocampal volume, and higher amyloid burden and p‐tau217 levels would amplify the effects of poor sleep quality on cognitive and functional decline. By clarifying the interaction between sleep quality and AD pathology in influencing clinical trajectories, this research aims to identify potential intervention targets across the spectrum of cognitive aging.

## 2 METHODS

### 2.1 Participants

Participants selected from the 1Florida ADRC (1FL ADRC) database included 326 participants at baseline (see Table 1 for follow‐up visits and demographic characteristics). The 1FL ADRC is an ongoing National Institute on Aging study that assesses the longitudinal cognitive and neurological changes in older individuals across the cognitive continuum from Florida who are either cognitively normal (CN) or have been diagnosed with MCI or dementia. Participants were recruited from three Florida sites: University of Miami, Mount Sinai Medical Center in Miami Beach, and University of Florida in Gainesville. Exclusion criteria included the presence of major psychiatric disturbances such as psychosis, bipolar, or unipolar disorders.

**TABLE 1 alz71470-tbl-0001:** Sample demographics and biomarker characteristics at baseline.

	Whole sample	CN	MCI	Dementia		
	*N = 326*	*n = 113*	*n = 192*	*n = 21*	*F* / *χ* ^2^	*p*
Age	66.35 (7.99)	64.74 (8.36)	66.57 (7.30)	72.95 (8.80)	2.04	<0.001
Education (years)	14.01 (3.37)	14.27 (3.12)	13.79 (3.56)	14.62 (2.73)	1.24	0.235
GDS	2.65 (2.68)	1.64 (2.07)	2.97 (2.79)	3.55 (2.79)	4.17	<0.001
CDR‐SB	1.35 (1.68)	0.09 (0.19)	1.59 (1.01)	5.93 (2.07)	—	—
Female, *n* (%)	165 (50.6%)	65 (57.5%)	86 (44.8%)	14 (66.7%)	7.37	0.025
Biomarkers						
Hippocampal volume (*n* = 227)	0.43 (0.00)	0.45 (0.01)	0.43 (0.00)	0.38 (0.02)	6.38	<0.001
p‐tau217 (*n* = 171)	0.39 (0.03)	0.31 (0.02)	0.42 (0.04)	0.79 (0.33)	12.20	<0.001
Amyloid CL (*n* = 133)	49.79 (3.54)	43.31 (4.40)	47.81 (4.23)	90.83 (22.1)	4.69	<0.001

*Notes*: Values are M (SD) unless otherwise indicated. F‐tests were used for continuous variables and *χ* ^2^ tests for categorical variables. CDR‐SB was used to determine diagnostic group membership and was not compared across groups.

Abbreviations: CDR‐SB, Clinical Dementia Rating scale Sum of Boxes; CL, Centiloid; CN, cognitively normal; F, F‐statistic; GDS, Geriatric Depression Scale; MCI, mild cognitive impairment; p‐tau, phosphorylated tau; SD, standard deviation; *χ* ^2^, chi‐squared.

### 2.2 Cognitive and functional diagnosis

The CDR‐SB is a widely used instrument for quantifying functional impairment in individuals suspected of having or diagnosed with neurodegenerative diseases, such as AD.^14^ The CDR assesses performance and capabilities in six domains: memory, orientation, judgment and problem solving, community affairs, home and hobbies, and personal care. Each of these domains is scored from 0 to 3, with higher scores indicating greater difficulty. The CDR‐SB score is calculated by summing all the domain scores obtained. Total scores range from 0 to 18, with 0 representing normal cognition and higher scores indicating increasing severity of functional impairment.

### 2.3 Depressive symptoms measures

Depressive symptoms were assessed using the 15‐item Geriatric Depression Scale (GDS‐15). The GDS is a widely used and validated self‐report instrument to screen for depression in older adult populations.^15^, ^16^ The scale uses a “yes/no” question format. Participants respond to 30 questions based on how they have felt over the past week, with each answer corresponding to a depressive response receiving 1 point. This yields a total score ranging from 0 to 15, with higher scores indicating greater depressive symptomatology.

**RESEARCH IN CONTEXT**

1. **Systematic review**: The authors reviewed the literature using PubMed, APA PsycNet, and Google Scholar. While associations among sleep duration, sleep timing, and Alzheimer's disease (AD) biomarkers (amyloid, tau, and hippocampal volume) have been documented cross‐sectionally, there is limited longitudinal research examining how these biomarkers moderate the relationship between sleep and cognitive/functional decline (Clinical Dementia Rating Sum of Boxes).
2. **Interpretation**: Our findings indicate that longer sleep duration and later wake times are markers of vulnerability in older adults. Critically, these sleep characteristics predict steeper cognitive and functional decline, specifically in individuals with existing AD pathology, such as elevated phosphorylated tau (p‐tau)217 and smaller hippocampal volumes.
3. **Future directions**: Future research should use objective sleep measures (e.g., actigraphy or polysomnography) to validate these subjective findings. Additionally, studies should investigate whether interventions targeting sleep timing can modify the clinical trajectory of individuals with high p‐tau217 or amyloid burden.

### 2.4 Sleep measures

Sleep quality was assessed using the PSQI, one of the most widely used subjective measurements of sleep quality, which has stronger reliability and validity compared to other sleep quality questionnaires.^17^, ^18^, ^19^ It has been accepted as a benchmark for assessing subjective sleep quality.^19^ The PSQI includes 24 items, of which 19 are self‐reported (included in scoring), and 5 are partner reported (unscored and only used for clinical purposes) questions about the participant's sleep habits. The questions are categorized into seven subsections: subjective sleep quality (C1), sleep latency (C2), sleep duration (C3), sleep efficiency (C4), sleep disturbances (C5), use of sleeping medication (C6), and daytime dysfunction (C7).

Each of these subcategories is scored from 0 (no difficulty) to 3 (severe difficulty), and the sum of these categories gives the total PSQI score, which ranges from 0 to 21. Higher scores indicate poorer sleep quality.

### 2.5 Brain biomarkers

Brain volumes were obtained using magnetic resonance imaging (MRI). Imaging data from the participants in this study were obtained using a Siemens Medical System Skyra 3 Tesla Scanner with software version “syngo MR E11” and coil “Siemens Head/Neck 20.” The scanning sequences used were 3D T1‐W magnetization‐prepared rapid gradient echo. Sagittal, three‐dimensional images with a resolution of 1 mm (≈ 12 minutes, repetition time = 2150 ms, echo time = 4.38 ms, inversion time = 1100 ms, 160 slices, 1×1×1 mm3) were obtained from ≈ 1 cm left of the skull to 1 cm right of the skull, allowing room for spatial reorientation along with defined anatomic coordinates. We examined the total volumes of the hippocampal brain regions from MRI scans.^20^

Aβ burden was determined through positron emission tomography (PET). Participants were scanned for 20 minutes on a Siemens Biograph 16 PET/computed tomography (CT) scanner operating in three‐dimensional mode (55 slices per frame, 3 mm slice thickness, 128 × 128 matrices). Amyloid load was measured continuously in Centiloids using a Siemens Biograph 16 PET/CT scanner in three‐dimensional mode (55 slices per frame, 3 mm slice thickness, 128 × 128 matrices) with the following tracer: NeuraCeq ([F‐18] florbetaben. Images included the top of the head to the top of the neck, and CT data were used to determine initial attenuation correction and image reconstruction in the coronal, sagittal, and axial planes. The florbetaben PET/CT was linear coregistered (i.e., trilinear interpolation) using 12 dfs to the T1 image; therefore, MRI parcellation and segmentation were the same as the florbetaben PET/CT image.^21^, [Table alz71470-tbl-0001]

### 2.6 Blood biomarkers

Blood samples were analyzed blinded to clinical and demographic data, using single‐molecule array (Simoa) technology for p‐tau217 (ALZpath). All samples had coefficients of variation < 20% (mean ± standard deviation 4.9 ± 3.8%) and good analytic performance (limit of detection = 0.001 pg/mL, lower limit of quantitation = 0.02 pg/mL, upper limit of quantitation = 10.0 pg/mL). Biomarkers were obtained at Visit 1. This blood biomarker was chosen because it is most closely associated with amyloid deposition on PET and is the most sensitive to AD.

### 2.7 Statistical analyses

Statistical analyses were performed using SPSS and R. Statistical significance was set at *p* < 0.05 unless otherwise specified. Assumptions for parametric tests were verified prior to analysis, including normality of distributions and homogeneity of variance where applicable. In all analyses, we controlled for age, education, and the presence of depressive symptoms. Sleep quality tends to decline with age,^22^ and years of education is one socioeconomic factor known to influence sleep patterns.^23^ Depression is highly prevalent in late life and strongly linked to cognitive decline, with studies showing it increases the risk of developing MCI and accelerating progression to dementia.^4^, ^8^, ^24^ While its role remains debated, either as an independent risk factor or an early manifestation of neurodegeneration, the consistent associations highlight depression as a critical determinant of cognitive aging trajectories.^4^, ^8^, ^25^, ^26^ Additionally, depression has been associated with subjective sleep disturbances.^27^

#### 2.7.1 Cross‐sectional analyses

In preliminary analyses, Pearson correlation coefficients were calculated to examine relationships between the seven PSQI subcomponents and CDR‐SB scores. To control for Type I error due to multiple comparisons, a Bonferroni correction was applied, adjusting the significance threshold to *p* < 0.007 (0.05/7). Components showing significant correlations were retained for subsequent regression analyses.

For primary regression models, hierarchical multiple linear regression analyses were conducted to examine sleep predictors of cognitive impairment (CDR‐SB). For each analysis, the model included age, years of education, GDS scores, and relevant sleep variables (PSQI components, global score, or sleep timing measures). False discovery rate (FDR) corrections were made to account for including several predictors and to avoid Type I errors.

In our biomarker interaction models, to test whether AD pathology and hippocampal volume moderate the relationship between sleep and CDR‐SB scores, hierarchical regression models were constructed including: (1) demographic covariates (age, education), (2) main effects of sleep variables and biomarkers (plasma p‐tau217, Centiloids, or hippocampal volume), and (3) sleep × biomarker interaction terms. FDR corrections were made to account for including several predictors and to avoid Type I errors.

### 2.8 Longitudinal analyses

Linear mixed‐effects models (LMMs) were used to investigate the longitudinal relationships between sleep variables and cognitive decline. Models included: (1) fixed effects: time (years from baseline), sleep variables (PSQI components, global score, or sleep timing), covariates (age, education, GDS), and relevant interactions; and (2) random effects: random intercepts for participants to account for individual differences in baseline CDR‐SB. FDR corrections were made to account for including several predictors and to avoid Type I errors.

Two‐way interactions (time × sleep) were tested to determine whether sleep variables predicted the rate of cognitive change. Three‐way interactions (time × sleep × biomarker) were examined to determine whether AD pathology or hippocampal volume moderated the relationship between sleep and cognitive trajectories. Maximum likelihood estimation was used for model comparison. FDR corrections were made to account for including several predictors and to avoid Type I errors.

## 3 RESULTS

### 3.1 Sample characteristics

Table 1 presents demographic and clinical characteristics across diagnostic groups at baseline. At baseline, the sample comprised 113 CN, 192 MCI, and 21 dementia participants. Groups differed significantly in age, with participants with dementia being the oldest, followed by those with MCI and CN. Depressive symptoms also differed across groups, with CN participants reporting fewer symptoms than participants with MCI or dementia. Education did not differ significantly across groups. Sample retention showed expected attrition, with 182 participants (55.8%) completing Visit 2 and 74 (22.7%) completing Visit 3 (see Table S1 in supporting information for demographics across all visits).

All regression and linear mixed models were initially run with sex as a covariate alongside age, education, and GDS. Across all analyses, including primary sleep–cognition models, biomarker interaction models, and longitudinal trajectories, sex was not a significant predictor of CDR‐SB (cross‐sectional models: all *p* > 0.10, range = 0.12–0.87; longitudinal models: all *p* > 0.15, range = 0.17–0.92). Additionally, sex did not significantly interact with any sleep parameters (PSQI components, wake time, sleep time) or AD biomarkers (p‐tau217, amyloid, hippocampal volume) in predicting cognitive outcomes. Given this consistent lack of association and following recommendations for model parsimony,^28^ sex was excluded from final models to preserve statistical power, particularly important given our biomarker subsample sizes and longitudinal attrition.

### 3.2 Sleep quality and cognitive impairment

#### 3.2.1 Cross‐sectional associations

Pearson correlations between PSQI subcomponents and CDR‐SB (Bonferroni‐corrected *α* = 0.007) revealed three significant associations: sleep duration (PSQI‐C3), for which higher scores indicated shorter sleep duration, negatively correlated with CDR‐SB (*r*[328] =−0.15, *p* = 0.006), while sleep disturbances (PSQI‐C5; *r*[270] = 0.18, *p* = 0.003) and daytime dysfunction (PSQI‐C7; *r*[324] = 0.19, *p* < 0.001) showed positive correlations.

A hierarchical regression with these three components revealed age (*B* = 0.058, *p* < 0.001), sleep duration (*B* =−0.203, *p* = 0.045), and GDS scores (*B* = 0.125, *p* < 0.001) remained significant predictors of CDR‐SB, while education and other sleep components were not significant.

Sleep onset time showed no association with CDR‐SB scores in the regression model (*p* = 0.852; Table S2 in supporting information). In contrast, later wake time significantly predicted worse CDR‐SB, accounting for 24% of variance (*p* = 0.002; Table S2).

#### 3.2.2 Longitudinal sleep effects

**FIGURE 1:**
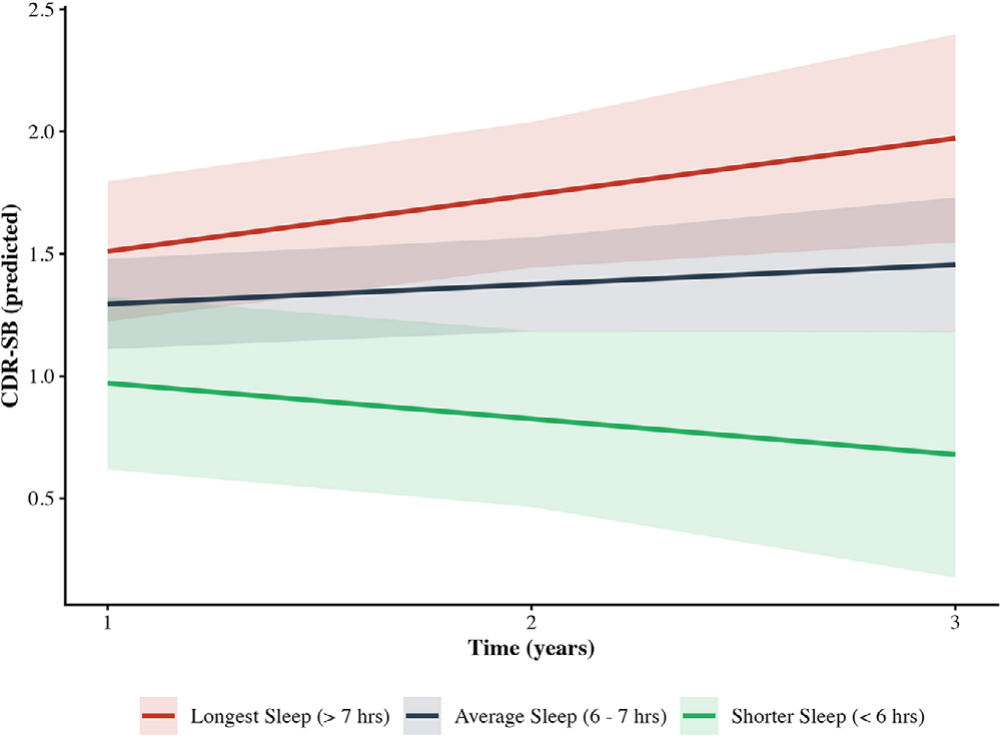
Sleep duration predicts trajectories of CDR‐SB. Predicted CDR‐SB scores over time as a function of baseline sleep duration (PSQI Component 3). Lines represent predicted values for longest sleep (> 7 hours; red), average sleep (6–7 hours; blue), and shorter sleep (< 6 hours; green). Shaded bands indicate 95% confidence intervals. Covariates (age, education, depressive symptoms [GDS]) held at sample means. Longer sleep duration was associated with worse CDR‐SB scores over time (time × sleep duration interaction: *B* =−0.15, P = 0.031). CDR‐SB, Clinical Dementia Rating Sum of Boxes; GDS, Geriatric Depression Scale; PSQI, Pittsburgh Sleep Quality Index.

Linear mixed models were used to examine sleep‐component predictors of CDR‐SB trajectories. The time × sleep duration interaction was significant (*B* =−0.17, 95% confidence interval [CI: −0.32, −0.02], *p* = 0.031; Table 2A; Figure 1), indicating participants with lower PSQI‐C3 scores (longer sleep duration) demonstrated steeper cognitive decline over time. The model demonstrated a good fit (marginal *R* ^2^ = 0.199, conditional *R* ^2^ = 0.739). Neither global sleep quality nor its interaction with time predicted CDR‐SB scores (Table S3 in supporting information). Wake time remained associated with worse overall cognition (*B* = 0.18, 95% CI [0.08, 0.28], *p* < 0.001) but did not interact with time to predict rate of decline (*p* = 0.910; Table 2B).

**TABLE 2:** Longitudinal mixed‐effects models: sleep characteristics predicting CDR‐SB.

Predictor	*df*1	*df*2	*F*	*B*	*SE*	*p*	FDR *p*	95% CI
*A. Sleep duration (PSQI‐C3) predicting CDR‐SB*								
Time	1	244.38	4.98	0.24	0.11	0.027	0.039	[0.03, 0.46]
PSQI‐C3	1	224.89	0.20	−0.06	0.13	0.654	0.654	[−0.32, 0.20]
Time × PSQI‐C3	1	242.85	4.73	−0.17	0.08	0.031	0.046	[−0.32, −0.02]
Age	1	306.42	32.30	0.06	0.01	<0.001	<0.001	[0.04, 0.08]
GDS	1	307.63	32.24	0.18	0.03	<0.001	<0.001	[0.12, 0.24]
*Marginal R* ^2^ * = 0.199, conditional R* ^2^ * = 0.739*								
*B. Wake time predicting CDR‐SB*								
Time	1	240.31	0.10	0.08	0.24	0.747	0.897	[−0.40, 0.56]
Wake time	1	288.77	12.51	0.18	0.05	<0.001	<0.001	[0.08, 0.28]
Time × Wake time	1	242.63	0.01	−0.004	0.04	0.910	0.909	[−0.07, 0.07]
Age	1	304.71	32.42	0.06	0.01	<0.001	<0.001	[0.04, 0.08]
GDS	1	305.36	26.63	0.15	0.03	<0.001	<0.001	[0.10, 0.21]
*Marginal R* ^2^ * = 0.208, conditional R* ^2^ * = 0.739*								

*Notes*: Denominator degrees of freedom are non‐integer values due to the Satterthwaite approximation, which accounts for the complex covariance structure of repeated measures data. Education included as covariate but not shown (all P > 0.05). Marginal R^2^ reflects variance explained by fixed effects; conditional R^2^ reflects variance explained by fixed and random effects.

Abbreviations: B, unstandardized coefficient; CDR‐SB, Clinical Dementia Rating scale Sum of Boxes; CI, confidence interval; df1, numerator degrees of freedom; df2, denominator degrees of freedom (Satterthwaite approximation); F, F‐statistic; FDR, false discovery rate; GDS, Geriatric Depression Scale; PSQI‐C3, Pittsburgh Sleep Quality Index, Component 3 (sleep duration); R^2^, coefficient of determination; SE, standard error.

### 3.3 AD biomarker and sleep interactions

#### 3.3.1 Cross‐sectional analyses

Higher p‐tau217 levels strongly predicted worse CDR‐SB scores. The wake time × p‐tau217 interaction was significant (*p* = 0.004; Table S4 in supporting information), indicating wake time effects on cognition were amplified in those with elevated p‐tau217. Sleep duration did not interact with p‐tau217 (*p* = 0.279; Table S4).

The sleep duration × amyloid burden interaction significantly predicted CDR‐SB (*B* = 0.011, *p* = 0.030; Table S4). Similarly, the wake time × amyloid interaction was significant (*p* = 0.006; Table S4).

Both sleep duration (*p* = 0.041; Table S4) and wake time (*p* = 0.004; Table S4) significantly interacted with hippocampal volume, suggesting increased sleep duration may impact CDR‐SB scores when hippocampal volume is smaller.

#### 3.3.2 Longitudinal analyses

Three‐way interactions revealed differential cognitive trajectories based on sleep and AD pathology. The time × sleep duration × p‐tau217 interaction was significant (*B* = –0.37, 95% CI [–0.71, –0.02], *P *= 0.036; Table 3A), as was time × wake time × p‐tau217 (*B* = 0.11, 95% CI [0.09, 0.14], *p* < 0.001; Table 3B). These findings suggest that the combination of poor sleep characteristics (later wake time and prolonged sleep duration) and elevated p‐tau217 levels accelerates cognitive/functional decline as measured by the CDR‐SB.

**TABLE 3:** Longitudinal mixed‐effects models: sleep × p‐tau217 interactions predicting CDR‐SB.

Predictor	*df*1	*df*2	*F*	*B*	*SE*	*p*	FDR *p*	95% CI
*A. Sleep duration × p‐tau217 predicting CDR‐SB*								
Time	1	191.88	2.12	0.18	0.13	0.147	0.196	[−0.07, 0.43]
PSQI‐C3	1	160.09	0.03	0.03	0.15	0.863	0.863	[−0.27, 0.32]
p‐tau217	1	164.19	62.68	1.21	0.15	<0.001	<0.001	[0.91, 1.51]
Time × PSQI‐C3	1	250.84	0.07	−0.03	0.10	0.791	0.791	[−0.23, 0.18]
Time × PSQI‐C3 × p‐tau217	1	186.82	4.44	−0.37	0.18	0.036	0.047	[−0.71, −0.02]
Age	1	162.32	14.55	0.04	0.01	<0.001	<0.001	[0.02, 0.06]
GDS	1	160.16	18.55	0.13	0.03	<0.001	<0.001	[0.07, 0.19]
*Marginal R* ^2^ * = 0.469, conditional R* ^2^ * = 0.658*								
*B. Wake time × p‐tau217 predicting CDR‐SB*								
Time	1	172.55	0.34	0.14	0.24	0.560	0.630	[−0.34, 0.62]
Wake time	1	241.90	0.22	0.03	0.07	0.639	0.639	[−0.10, 0.17]
*p*‐tau217	1	160.42	4.75	−1.84	0.85	0.031	0.052	[−3.51, −0.17]
Wake Time × *p*‐tau217	1	172.34	4.64	0.25	0.12	0.033	0.049	[0.02, 0.47]
Time × Wake × *p*‐tau217	1	135.86	71.26	0.11	0.01	<0.001	<0.001	[0.09, 0.14]
Age	1	163.35	16.17	0.04	0.01	<0.001	<0.001	[0.02, 0.06]
GDS	1	160.87	20.64	0.13	0.03	<0.001	<0.001	[0.07, 0.19]
*Marginal R* ^2^ * = 0.575, conditional R* ^2^ * = 0.759*								

*Notes*: Denominator degrees of freedom are non‐integer values due to the Satterthwaite approximation, which accounts for the complex covariance structure of repeated measures data. Education included as covariate but not shown (all P > 0.05). Marginal R^2^ reflects variance explained by fixed effects; conditional R^2^ reflects variance explained by fixed and random effects.

Abbreviations: B, unstandardized coefficient; CDR‐SB, Clinical Dementia Rating scale Sum of Boxes; CI, confidence interval; df1, numerator degrees of freedom; df2, denominator degrees of freedom (Satterthwaite approximation); F, F‐statistic; FDR, false discovery rate; GDS, Geriatric Depression Scale; PSQI‐C3, Pittsburgh Sleep Quality Index, Component 3 (sleep duration); p‐tau217, phosphorylated tau‐217; R^2^, coefficient of determination; SE, standard error.

For amyloid, the three‐way time × wake time × amyloid interaction was significant (*B* = 0.003, 95% CI [0.002, 0.004], *p* < 0.001; Table 4B) with individuals showing both later wake times and higher amyloid burden demonstrating the steepest CDR‐SB increases. When examining sleep duration and Aβ levels’ impact on CDR‐SB over time, the sleep duration × amyloid × time interaction was not significant (*p* = 0.789; Table 4A).

**TABLE 4:** Longitudinal mixed‐effects models: sleep × amyloid interactions predicting CDR‐SB.

Predictor	*df*1	*df*2	*F*	*B*	*SE*	*p*	FDR *p*	95% CI
*A. Sleep duration × amyloid predicting CDR‐SB*								
Time	1	83.72	11.43	0.75	0.22	0.001	0.003	[0.31, 1.19]
PSQI‐C3	1	67.44	0.61	0.20	0.25	0.437	0.466	[−0.31, 0.71]
Amyloid	1	140.68	17.40	0.02	0.004	<0.001	<0.001	[0.01, 0.03]
Time × PSQI‐C3	1	102.12	4.92	−0.46	0.21	0.029	0.048	[−0.88, −0.05]
Time × PSQI‐C3 × amyloid	1	88.21	0.07	0.001	0.002	0.789	0.789	[−0.003, 0.004]
Age	1	128.07	2.05	0.03	0.02	0.154	0.205	[−0.01, 0.07]
GDS	1	128.17	11.63	0.20	0.06	<0.001	0.002	[0.08, 0.31]
*Marginal R* ^2^ * = 0.282, conditional R* ^2^ * = 0.987*								
*B. Wake time × amyloid predicting CDR‐SB*								
Time	1	73.14	1.15	−0.42	0.40	0.287	0.385	[−1.21, 0.36]
Wake time	1	208.77	0.62	0.07	0.09	0.434	0.463	[−0.11, 0.24]
Amyloid	1	134.00	0.74	−0.01	0.01	0.390	0.439	[−0.02, 0.01]
Time × wake time	1	76.93	0.38	−0.04	0.06	0.537	0.537	[−0.15, 0.08]
Wake time × amyloid	1	193.32	0.13	0.000	0.001	0.716	0.716	[−0.002, 0.001]
Time × wake × amyloid	1	59.32	35.97	0.003	0.0005	<0.001	<0.001	[0.002, 0.004]
Age	1	134.00	2.86	0.03	0.02	0.093	0.140	[−0.01, 0.07]
GDS	1	134.00	12.26	0.18	0.05	<0.001	0.003	[0.08, 0.27]
*Marginal R* ^2^ * = 0.405, conditional R* ^2^ * = 0.810*								

*Notes*: Denominator degrees of freedom are non‐integer values due to the Satterthwaite approximation, which accounts for the complex covariance structure of repeated measures data. Amyloid measured via PET in Centiloid units. Education included as covariate but not shown (all P > 0.05). Marginal R^2^ reflects variance explained by fixed effects; conditional R^2^ reflects variance explained by fixed and random effects.

Abbreviations: B, unstandardized coefficient; CDR‐SB, Clinical Dementia Rating scale Sum of Boxes; CI, confidence interval; df1, numerator degrees of freedom; df2, denominator degrees of freedom (Satterthwaite approximation); F, F‐statistic; FDR, false discovery rate; GDS, Geriatric Depression Scale; PET, positron emission tomography; PSQI‐C3, Pittsburgh Sleep Quality Index, Component 3 (sleep duration); R^2^, coefficient of determination; SE, standard error.

**FIGURE 2:**
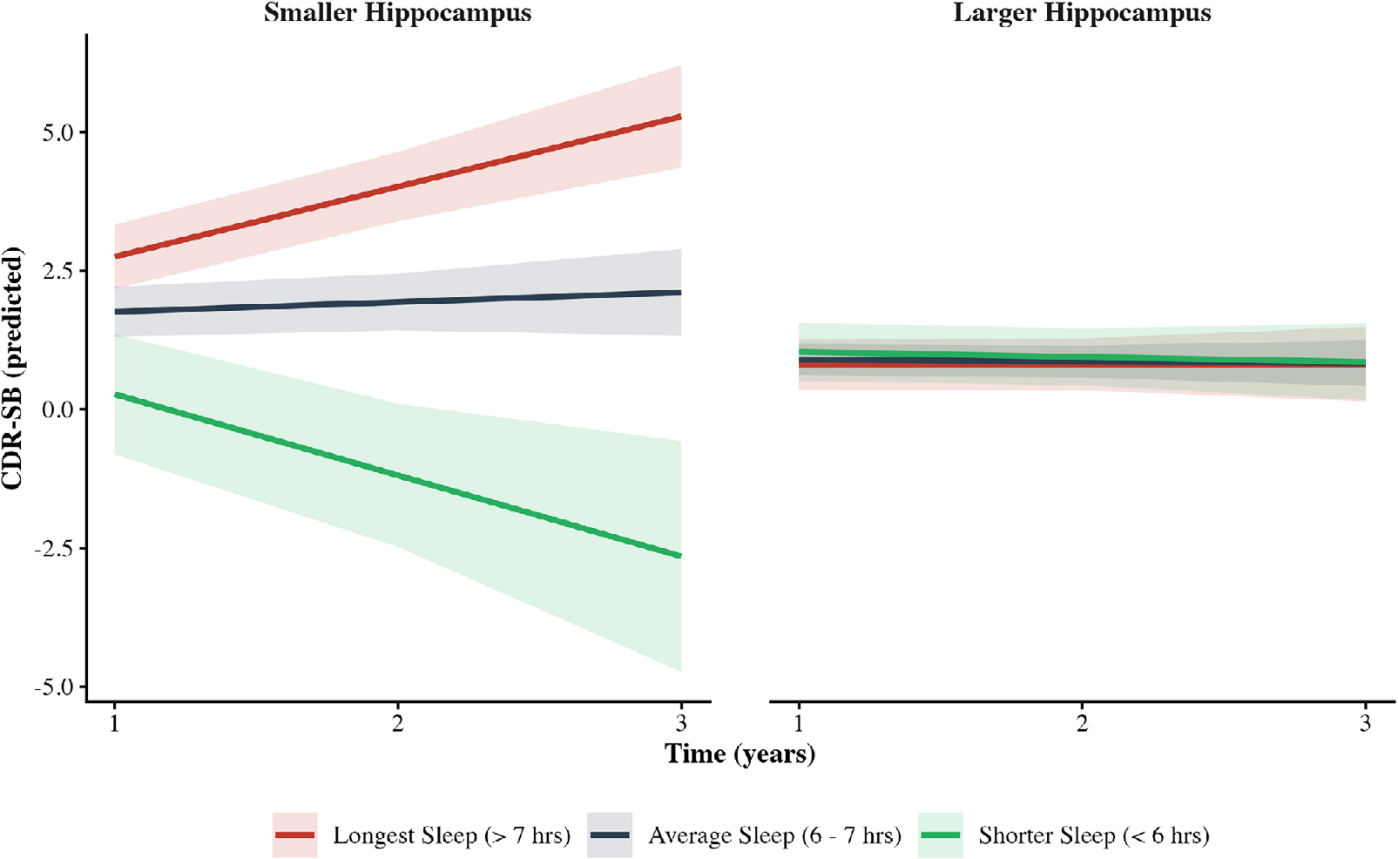
Sleep duration × hippocampal volume interaction predicting CDR‐SB trajectories. Predicted CDR‐SB scores over time as a function of baseline sleep duration (PSQI Component 3) and hippocampal volume. Panels display predicted trajectories for individuals with smaller hippocampal volume (left) and larger hippocampal volume (right). Lines represent predicted values for longest sleep (> 7 hours; red), average sleep (6–7 hours; blue), and shorter sleep (< 6 hours; green). The CDR‐SB scale ranges from 0 to 18, with higher scores indicating greater impairment. Negative predicted values in the shorter sleep/smaller hippocampus condition represent statistical extrapolation from the linear mixed model; clinically, these values would correspond to scores at or near the floor of 0 (no impairment). Sleep duration categories were based on PSQI Component 3 scoring: 0 = > 7 hours, 1 = 6–7 hours, and 2.5 = < 6 hours (representing the midpoint of scores 2 [5–6 hours] and 3 [< 5 hours]). Hippocampal volume categories were derived from the sample mean (M = 0.419) ± 1 SD (0.058), yielding smaller hippocampal volume (0.361, −1 SD) and larger hippocampal volume (0.477, +1 SD). Shaded bands indicate 95% confidence intervals. Covariates (age, education, depressive symptoms) were held at sample means. Among individuals with larger hippocampal volume, sleep duration had minimal impact on CDR‐SB trajectories; all sleep groups showed stable, low CDR‐SB scores over time. In contrast, among individuals with smaller hippocampal volume, longer sleep duration was associated with markedly faster cognitive decline, while shorter sleepers showed more modest changes. This pattern suggests that the combination of longer sleep duration and reduced hippocampal volume, a marker of neurodegeneration, is associated with particularly accelerated worsening CDR‐SB scores. The three‐way time × sleep duration × hippocampal volume interaction was significant, *F*(1, 318.74) = 11.63, *p* < 0.001. CDR‐SB, Clinical Dementia Rating Sum of Boxes; PSQI, Pittsburgh Sleep Quality Index; SD, standard deviation.

**FIGURE 3:**
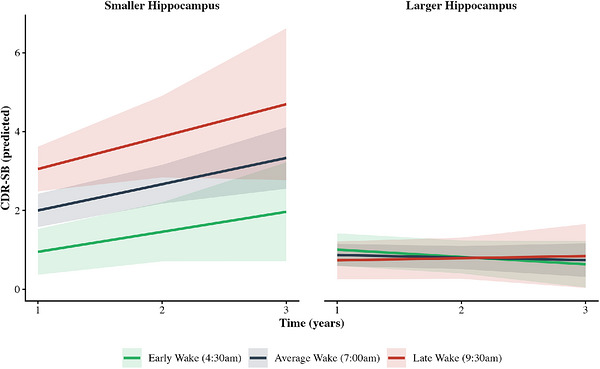
Wake time × hippocampal volume interaction predicting CDR‐SB trajectories. Predicted CDR‐SB scores over time as a function of wake time and hippocampal volume. Panels display predicted trajectories for individuals with smaller hippocampal volume (left) and larger hippocampal volume (right). Lines represent predicted values for early wake time (4:30 am; green), average wake time (7:00 am; blue), and late wake time (9:30 am; red). Wake time categories were derived from the sample mean (*M* = 6:53 am) ± 1 SD (2.5 hours), yielding early wake (4:30 am, −1 SD), average wake (7:00 am, mean), and late wake (9:30 am, +1 SD). Hippocampal volume categories were derived from the sample mean (*M* = 0.419) ± 1 SD (0.058), yielding smaller hippocampal volume (0.361, −1 SD) and larger hippocampal volume (0.477, +1 SD). Shaded bands indicate 95% confidence intervals. Covariates (age, education, depressive symptoms) held at sample means. Among individuals with larger hippocampal volume, wake time had minimal impact on CDR‐SB trajectories; all groups showed stable, low CDR‐SB scores over time. In contrast, among individuals with smaller hippocampal volume, later wake time was associated with higher baseline CDR‐SB and faster decline, while early risers showed more stable trajectories. This pattern suggests that the combination of later wake time and reduced hippocampal volume is associated with accelerated worsening CDR‐SB score. The three‐way time × wake time × hippocampal volume interaction was significant, *F*(1, 174.37) = 12.30, *p* < 0.001. CDR‐SB, Clinical Dementia Rating Sum of Boxes; SD, standard deviation.

Hippocampal volume showed significant three‐way interactions with both sleep duration (*B* = 5.24, 95% CI [2.22, 8.27], *p* < 0.001; Table 5A; Figure 2) and wake time (*B* =−1.03, 95% CI [−1.61, −0.45], *p* < 0.001; Table 5B; Figure 3). Participants with both later wake times and longer sleep durations, who had smaller hippocampal volumes, showed the most pronounced cognitive decline.

**TABLE 5:** Longitudinal mixed‐effects models: sleep × hippocampal volume interactions predicting CDR‐SB.

Predictor	*df*1	*df*2	*F*	*B*	*SE*	*p*	FDR *p*	95% CI
*A. Sleep duration × hippocampal volume predicting CDR‐SB*								
Time	1	180.66	10.58	0.47	0.15	0.001	0.004	[0.19, 0.76]
PSQI‐C3	1	181.10	1.54	0.21	0.17	0.217	0.248	[−0.13, 0.55]
Hippocampal volume	1	265.11	36.83	−15.20	2.51	<0.001	<0.001	[−20.14, −10.27]
Time × PSQI‐C3	1	324.43	14.11	−2.74	0.73	<0.001	<0.001	[−4.18, −1.31]
Time × PSQI‐C3 × hippocampus	1	318.74	11.63	5.24	1.54	<0.001	<0.001	[2.22, 8.27]
Age	1	219.15	3.88	0.03	0.01	0.050	0.067	[0.00, 0.05]
GDS	1	218.85	12.77	0.14	0.04	<0.001	<0.001	[0.06, 0.21]
*Marginal R* ^2^ * = 0.297, conditional R* ^2^ * = 0.722*								
*B. Wake time × hippocampal volume predicting CDR‐SB*								
Time	1	174.64	0.69	−0.26	0.31	0.407	0.407	[−0.88, 0.36]
Wake time	1	293.87	13.63	1.38	0.38	<0.001	<0.001	[0.65, 2.12]
Hippocampal volume	1	219.10	8.92	17.87	5.99	0.003	0.003	[6.08, 29.67]
Time × wake time	1	173.98	13.27	0.51	0.14	<0.001	<0.001	[0.24, 0.79]
Wake time × hippocampus	1	277.29	12.29	−3.04	0.87	<0.001	<0.001	[−4.74, −1.33]
Time × wake × hippocampus	1	174.37	12.30	−1.03	0.29	<0.001	<0.001	[−1.61, −0.45]
Age	1	220.86	3.28	0.02	0.01	0.072	0.072	[−0.002, 0.05]
GDS	1	220.52	15.77	0.14	0.04	<0.001	<0.001	[0.07, 0.21]
*Marginal R* ^2^ * = 0.282, conditional R* ^2^ * = 0.732*								

*Notes*: Denominator degrees of freedom are non‐integer values due to the Satterthwaite approximation, which accounts for the complex covariance structure of repeated measures data. Hippocampal volume expressed as a proportion of total intracranial volume. Education included as covariate but not shown (all P > 0.05). Marginal R^2^ reflects variance explained by fixed effects; conditional R^2^ reflects variance explained by fixed and random effects.

**Abbreviations**: B, unstandardized coefficient; CDR‐SB, Clinical Dementia Rating scale Sum of Boxes; CI, confidence interval; df1, numerator degrees of freedom; df2, denominator degrees of freedom (Satterthwaite approximation); F, F‐statistic; FDR, false discovery rate; GDS, Geriatric Depression Scale; PSQI‐C3, Pittsburgh Sleep Quality Index, Component 3 (sleep duration); R^2^, coefficient of determination; SE, standard error.

## 4 DISCUSSION

This study examined whether subjective sleep characteristics (using the PSQI) are associated with cognitive/functional decline in older adults, and whether AD biomarkers moderate these relationships. Our findings suggest that prolonged sleep duration and delayed wake timing may reflect an underlying vulnerability to neurodegeneration. Critically, the impact of sleep disturbances on cognitive trajectories depended on AD pathological burden: individuals with elevated p‐tau217, higher amyloid levels, or reduced hippocampal volume were most susceptible to the detrimental effects of sleep characteristics. These findings have implications for how we conceptualize sleep disturbances in the context of MCI and dementia, giving insight into who may be at greater risk for accelerated decline.

At baseline, longer sleep duration was associated with worse cognitive/functional performance. Further longitudinal analyses also revealed that longer sleep duration predicted progressively steeper CDR‐SB scores over time. This finding requires careful interpretation. Rather than suggesting that sleep itself is harmful, these results are likely to reflect reverse causation or unmeasured factors related to sleep quality. Long sleep duration in older adults often indicates fragmented, inefficient sleep rather than restorative rest.^29^ This supports past literature which established that prolonged sleep durations are significantly associated with indicators of impairment and neurodegeneration, with studies observing distinct risks related to both short and long sleep relative to diagnoses such as MCI and dementia.^30^, ^31^ In larger cohort studies, sleeping ≤ 4 hours per night was associated with significantly lower baseline global cognitive function scores and a faster rate of global cognitive decline longitudinally, compared to the reference group (7 hours).^29^ Conversely, prolonged or long sleep duration (typically defined as ≥ 9 hours or ≥ 10 hours) is often associated with cognitive impairment, both cross‐sectionally and longitudinally, including being associated with an increased risk of incident dementia^12^, ^30^, ^31^, ^32^ and leading to a faster rate of global cognitive decline compared to moderate sleepers.^29^ Additionally, prolonged sleep duration has been found to be generally longer in participants with MCI compared to those without it.^33^ Furthermore, a duration exceeding 8 hours increased the odds of having MCI in literate older adults in one study.^34^

Longer sleep in late life may not be protective but instead may signal underlying neurodegenerative processes or medical comorbidities. Although adequate sleep is essential for brain health, evidence suggests that prolonged sleep in late life is more often a marker of vulnerability than a protective factor^12^, ^30^ ^−32^. While it is often accepted that longer sleep could confer benefits, this seems less applicable to older adults, for whom extended sleep duration could be characterized by fragmentation and reduced efficiency. Large cohort studies in older adults have consistently associated ≥ 9 hours of sleep with a greater risk of dementia, accelerated cognitive decline, and reduced brain volumes, even after adjusting for comorbidities.^32^, ^35^ These findings suggest that excessive sleep may be associated with underlying neurodegenerative processes or related health conditions, rather than serving as a compensatory or protective mechanism.^12^, ^36^

Our results demonstrated that wake time had a consistent and independent relationship with CDR‐SB scores. Later wake times were associated with greater cognitive and functional impairment at baseline, even after adjusting for age, education, and depressive symptoms. This finding suggests that the timing of awakening, rather than the timing of sleep initiation, may be more closely linked to cognitive function. There are several possible explanations. Later wake times may indicate a delay in the circadian rhythm or a reduction in circadian amplitude, both of which are associated with neurodegeneration and have been reported in individuals with MCI and AD.^37^ Also, waking later may reflect extended time in bed, which aligns with our other findings that longer sleep is associated with increased cognitive and functional decline over time.

Across regression models, p‐tau217 concentration emerged as a robust and independent predictor of cognitive impairment, accounting for substantial variance in CDR‐SB. This finding aligns with prior studies that have identified p‐tau as a strong biomarker of AD progression and clinical severity.^12^ In contrast, sleep duration and daytime dysfunction did not independently predict cognition once biomarkers were considered, nor did they significantly interact with p‐tau217 cross‐sectionally. This suggests that while subjective sleep complaints are associated with cognition in simpler models, their predictive utility is largely overshadowed by AD biomarkers when considered simultaneously.

When amyloid burden was examined, a different pattern emerged. The interaction between sleep duration and amyloid burden predicted CDR‐SB scores cross‐sectionally. The detrimental effect of longer sleep on the CDR‐SB score was amplified in individuals with higher amyloid load. This finding supports models of AD pathology in which diminished sleep quality and amyloid deposition jointly accelerate clinical impairment, possibly through impaired glymphatic clearance or enhanced amyloid accumulation during fragmented sleep.^12^

Hippocampal volume showed a moderating effect: lower volume and its interaction with sleep duration predicted worse CDR‐SB scores, indicating that longer sleep is more detrimental in individuals with smaller hippocampi. In contrast, a larger hippocampal volume appeared protective, supporting the concept of neural reserve and aligning with prior findings that link hippocampal atrophy to accelerated cognitive decline in aging.^7^, ^22^, ^38^

Cross‐sectionally, later wake times were consistently associated with worse CDR‐SB scores, particularly in individuals with greater AD pathology. Wake time interacted with p‐tau217 and amyloid burden, such that delayed waking was more strongly linked to poorer CDR‐SB scores among those with higher tau or amyloid levels. Wake time also interacted with hippocampal volume, with the strongest associations observed in individuals with smaller hippocampi. This pattern aligns with prior findings linking nighttime behaviors, cognitive impairment, and hippocampal atrophy.^7^ Overall, later wake times appear especially relevant to cognitive and functional impairment in individuals with elevated AD pathology and reduced hippocampal volume.

Longitudinal models clarified how sleep interacts with AD pathology and brain structure over time. P‐tau217 showed a strong, stable association with cognitive/functional decline, and this effect was moderated by sleep duration: individuals with longer sleep and higher p‐tau217 exhibited the steepest increases in CDR‐SB. In contrast, although longer sleep predicted faster decline in the amyloid model, this effect was independent of amyloid burden, as the three‐way interaction (time × sleep duration × amyloid) was not significant. This differs from the p‐tau217 findings and suggests that, while long sleep and high amyloid are associated with worse cross‐sectional CDR‐SB scores, they may not drive accelerated future decline. Instead, longitudinal results suggest that p‐tau pathology has a more pronounced interaction with sleep duration, accelerating cognitive decline. Finally, hippocampal volume moderated the relationship between sleep duration and CDR‐SB over time: longer sleep predicted greater decline over time, particularly in individuals with smaller hippocampi, supporting prior work identifying hippocampal atrophy as a key AD biomarker.^38^

In longitudinal analyses, wake timing alone predicted decline in CDR‐SB over time, as well as remained significant when considered in conjunction with tau pathology. The significant three‐way interaction among time, wake time, and plasma p‐tau217 suggests that later wake timing is particularly detrimental in individuals with higher tau burden. In participants with elevated p‐tau217, later wake times were associated with steeper increases in CDR‐SB scores over time, whereas this pattern was not observed in those with lower tau. These results imply that later wake timing may reflect tau‐related disruption of circadian regulation or neural network integrity, rather than being an independent cognitive risk factor. Aligning with the wake time and p‐tau217 findings, the interaction between wake time and amyloid burden with time was significant, indicating that greater Aβ burden amplified the association between sleep quality and CDR‐SB scores over time. Last, a smaller hippocampal volume also significantly interacted with later wake time to predict worse CDR‐SB scores over time.

Some limitations should be considered when interpreting these findings. Sleep quality was assessed using the PSQI, a self‐report measure subject to recall bias, particularly among individuals with memory impairment; future studies should incorporate objective measures such as polysomnography or wearable devices. Although CDR‐SB was collected longitudinally, AD biomarkers were measured at a single time point, precluding analyses of longitudinal sleep–biomarker trajectories. The PSQI does not assess daytime napping, and sleep medication use was not examined, both of which may influence cognitive outcomes. Years of education served as a proxy for socioeconomic status; although widely used in epidemiological research, it may not fully capture the complexity of socioeconomic factors. Additionally, the modest size of the dementia subsample may limit the precision of estimates for this subgroup. Finally, although the sample included participants from three Florida sites within the 1FL ADRC group, external validation in independent cohorts is needed to confirm generalizability across broader populations and clinical settings.

## CONFLICT OF INTEREST STATEMENT

The authors declare no conflicts of interest. Author disclosures are available in the Supporting Information.

## CONSENT STATEMENT

All participants provided informed consent.

## Supporting information




**Supporting Information**: alz71470‐sup‐0001‐tablesS1‐S4.docx

**Supporting Information**:alz71470‐sup‐0002‐SuppMat.pdf
