# Supplementary material for: Associations among sleep quality, cognitive decline, and Alzheimer's disease pathology in older adults: A longitudinal study
Source: Alzheimers Dement. 2026 Jun 3;22(6):e71470. doi: 10.1002/alz.71470 (PMC13238871; doi:10.1002/alz.71470)
Supplement: Supplementary file 1 — Supporting Information: alz71470‐sup‐0001‐tablesS1‐S4.docx [file ALZ-22-e71470-s001.docx]

**SUPPLEMENTARY TABLES**

**Supplementary Table S1.** *Sample Demographics by Visit and Diagnostic Group*

|  | **Whole Sample** | **CN** | **MCI** | **Dementia** | ***F / χ²*** | ***p*** | **η** |
| --- | --- | --- | --- | --- | --- | --- | --- |
| ***Visit 1*** | | | | | | | |
|  | *n = 326* | *n = 113* | *n = 192* | *n = 21* |  |  |  |
| Age | 66.35 (7.99) | 64.74 (8.36) | 66.57 (7.30) | 72.95 (8.80) | 2.04 | <.001 | .221 |
| Education | 14.01 (3.37) | 14.27 (3.12) | 13.79 (3.56) | 14.62 (2.73) | 1.24 | .235 | .060 |
| GDS | 2.65 (2.68) | 1.64 (2.07) | 2.97 (2.79) | 3.55 (2.79) | 4.17 | <.001 | .159 |
| CDR-SB | 1.35 (1.68) | 0.09 (0.19) | 1.59 (1.01) | 5.93 (2.07) | — | — | — |
| Female, n (%) | 165 (50.6%) | 65 (57.5%) | 86 (44.8%) | 14 (66.7%) | 7.37 | .025 | — |
| ***Visit 2*** | | | | | | | |
|  | *n = 182* | *n = 79* | *n = 92* | *n = 11* |  |  |  |
| Age | 66.68 (7.54) | 64.94 (7.91) | 67.49 (7.91) | 72.36 (7.26) | 1.73 | .015 | .278 |
| Education | 13.66 (3.00) | 13.48 (2.85) | 13.50 (3.03) | 16.36 (2.66) | 3.74 | .026 | .040 |
| GDS | 2.51 (2.54) | 1.67 (2.07) | 2.97 (2.61) | 3.25 (2.83) | 1.13 | <.001 | .222 |
| CDR-SB | 1.12 (1.87) | 0.08 (0.18) | 1.34 (0.94) | 6.77 (3.30) | — | — | — |
| Time from V1 (mo) | 12.96 (2.58) | 12.70 (2.36) | 12.99 (2.63) | 14.73 (3.40) | 2.53 | .083 | .027 |
| Female, n (%) | 97 (53.3%) | 41 (51.9%) | 47 (51.1%) | 9 (81.8%) | 3.84 | .147 | — |
| ***Visit 3*** | | | | | | | |
|  | *n = 74* | *n = 38* | *n = 28* | *n = 8* |  |  |  |
| Age | 67.22 (7.43) | 65.32 (6.64) | 67.89 (7.71) | 75.86 (4.30) | 5.25 | <.001 | .780 |
| Education | 13.25 (3.25) | 13.13 (3.16) | 13.00 (3.45) | 14.57 (3.41) | 0.62 | .807 | .117 |
| GDS | 2.14 (2.39) | 1.46 (1.95) | 2.42 (2.38) | 2.28 (2.75) | 3.13 | .003 | .403 |
| CDR-SB | 0.99 (1.87) | 0.03 (0.11) | 1.17 (0.73) | 5.79 (1.28) | — | — | — |
| Time from V2 (mo) | 12.48 (2.80) | 12.33 (3.06) | 12.74 (2.64) | 12.63 (1.83) | 0.89 | .417 | .029 |
| Female, n (%) | 34 (46.0%) | 17 (44.7%) | 13 (46.4%) | 4 (50.0%) | 0.68 | .711 | — |

**Notes:** Values are M (SD). F-tests were used for continuous variables and χ² tests for categorical variables. CDR-SB was used to determine diagnostic group membership

and was not compared across groups.

**Abbreviations:** CDR-SB, Clinical Dementia Rating Scale-Sum of Boxes; CN, cognitively normal; F, F-statistic; GDS, Geriatric Depression Scale; MCI, mild cognitive

impairment; η, eta (measure of effect size); χ², chi-squared.

**Supplementary Table S2.** *Cross-Sectional Regression: Sleep Characteristics Predicting CDR-SB*

| **Variable** | **B** | **95% CI** | **β** | **t** | ***p*** | **FDR *p*** |
| --- | --- | --- | --- | --- | --- | --- |
| ***Predictor: Sleep Time (R² = .171)*** | | | | | | |
| Age | 0.062 | [0.041, 0.084] | .297 | 5.68 | <.001 | <.001 |
| Education | -0.034 | [-0.086, 0.018] | -.070 | -1.30 | .194 | .222 |
| GDS | 0.163 | [0.102, 0.225] | .275 | 5.23 | <.001 | .001 |
| Sleep Time | 0.003 | [-0.025, 0.030] | .010 | 0.19 | .852 | .852 |
| ***Predictor: Wake Time (R² = .240)*** | | | | | | |
| Age | 0.057 | [0.036, 0.077] | .272 | 5.42 | <.001 | <.001 |
| Education | -0.046 | [-0.095, 0.004] | -.093 | -1.81 | .072 | .096 |
| GDS | 0.143 | [0.085, 0.202] | .244 | 4.84 | <.001 | <.001 |
| Wake Time | 0.179 | [0.114, 0.245] | .267 | 5.37 | <.001 | .002 |
| **Notes:** Values are unstandardized unless otherwise noted.  **Abbreviations:** B, unstandardized coefficient; CDR-SB, Clinical Dementia Rating Scale-Sum of Boxes; CI, confidence interval; FDR, false discovery rate; GDS, Geriatric Depression Scale; R², coefficient of determination; t, t-statistic; β, standardized coefficient. | | | | | | |

**Supplementary Table S3.** *Longitudinal: PSQI Global Score Predicting CDR-SB*

| **Predictor** | **df1** | **df2** | **F** | **B** | **SE** | ***p*** | **FDR *p*** | **95% CI** |
| --- | --- | --- | --- | --- | --- | --- | --- | --- |
| ***PSQI Global Predicting CDR-SB (R²m = .218, R²c = .742)*** | | | | | | | | |
| Time | 1 | 172.2 | 0.72 | 0.12 | .14 | .398 | .478 | [-0.16, 0.39] |
| PSQI Global | 1 | 237.0 | 0.09 | -0.01 | .03 | .764 | .764 | [-0.08, 0.06] |
| Time × PSQI Global | 1 | 180.0 | 0.86 | -0.02 | .02 | .355 | .478 | [-0.05, 0.02] |
| Age | 1 | 250.1 | 35.61 | 0.07 | .01 | <.001 | <.001 | [0.05, 0.09] |
| GDS | 1 | 249.8 | 33.04 | 0.19 | .03 | <.001 | <.001 | [0.13, 0.26] |

**Notes:** Denominator degrees of freedom are non-integer values due to the Satterthwaite approximation, which accounts for the complex covariance structure of repeated

measures. Education included as covariate but not shown (p = .564).

**Abbreviations:** B, unstandardized coefficient; CDR-SB, Clinical Dementia Rating Scale-Sum of Boxes; CI, confidence interval; df1, numerator degrees of freedom; df2,

denominator degrees of freedom (Satterthwaite approximation); F, F-statistic; FDR, false discovery rate; GDS, Geriatric Depression Scale; PSQI, Pittsburgh Sleep Quality Index;

R²c, conditional R-squared; R²m, marginal R-squared; SE, standard error.

**Supplementary Table S4.** *Cross-Sectional: Sleep × Biomarker Interactions Predicting CDR-SB*

| **Variable** | **B** | **95% CI** | **β** | **t** | ***p*** | **FDR *p*** |
| --- | --- | --- | --- | --- | --- | --- |
| ***Sleep Duration × p-tau217 (R² = .354)*** | | | | | |  |
| PSQI-C3 | -0.010 | [-0.306, 0.285] | -.008 | -0.07 | .945 | .945 |
| p-tau217 | 0.949 | [0.617, 1.280] | .394 | 5.66 | <.001 | .002 |
| PSQI-C3 × p-tau217 | -0.484 | [-1.205, 0.236] | -.140 | -1.33 | .186 | .279 |
| ***Wake Time × p-tau217 (R² = .379)*** | | | | | |  |
| Wake Time | -0.037 | [-0.139, 0.066] | -.064 | -0.71 | .480 | .640 |
| p-tau217 | -1.910 | [-3.682, -0.138] | -.849 | -2.13 | .035 | .060 |
| Wake Time × p-tau217 | 0.373 | [0.137, 0.609] | 1.264 | 3.13 | .002 | .004 |
| ***Sleep Duration × Amyloid (R² = .296)*** | | | | | |  |
| PSQI-C3 | 0.319 | [-0.239, 0.878] | .134 | 1.13 | .260 | .347 |
| Amyloid | -0.007 | [-0.026, 0.013] | -.144 | -0.70 | .486 | .530 |
| PSQI-C3 × Amyloid | 0.011 | [0.003, 0.020] | .615 | 2.62 | .010 | .030 |
| ***Wake Time × Amyloid (R² = .408)*** | | | | | |  |
| Wake Time | 0.034 | [-0.103, 0.171] | .050 | 0.49 | .624 | .624 |
| Amyloid | -0.006 | [-0.020, 0.008] | -.126 | -0.84 | .403 | .484 |
| Wake Time × Amyloid | 0.003 | [0.001, 0.004] | .606 | 3.44 | <.001 | .006 |
| ***Sleep Duration × Hippocampus (R² = .233)*** | | | | | |  |
| PSQI-C3 | -0.186 | [-0.420, 0.048] | -.097 | -1.57 | .118 | .156 |
| Hippocampus | -66.225 | [-114.99, -17.46] | -1.991 | -2.68 | .008 | .016 |
| PSQI-C3 × Hippocampus | 64.707 | [8.51, 120.91] | 1.678 | 2.27 | .024 | .041 |
| ***Wake Time × Hippocampus (R² = .336)*** | | | | | |  |
| Wake Time | 1.897 | [1.202, 2.592] | .271 | 5.38 | <.001 | .004 |
| Hippocampus | -17.816 | [-29.76, -5.87] | -.533 | -2.94 | .004 | .010 |
| Wake × Hippocampus | -4.070 | [-5.70, -2.44] | -.271 | -4.93 | <.001 | .004 |

**Notes.** Values are unstandardized unless otherwise noted. Age, education, and GDS were included as covariates but not shown.

**Abbreviations:** B, unstandardized coefficient; CDR-SB, Clinical Dementia Rating Scale-Sum of Boxes; CI, confidence interval; FDR, false discovery rate; GDS, Geriatric

Depression Scale; PSQI-C3, Pittsburgh Sleep Quality Index, Component 3 (Sleep Duration); R², coefficient of determination; t, t-statistic; β, standardized coefficient.
